# 10.6-μm infrared laser as adjuvant therapy for diabetic peripheral neuropathy: study protocol for a double-blind, randomized controlled trial

**DOI:** 10.1186/s13063-021-05901-6

**Published:** 2022-01-18

**Authors:** Lin Lin, Yi Chen, Yuxia Li, Ke Cheng, Haiping Deng, Jianping Lu, Ling Zhao, Xueyong Shen

**Affiliations:** 1 School of Nursing, Shanghai University of Traditional Chinese Medicine, 1200 Cailun Road, Shanghai, 201203 China; 2 Shanghai Municipal Hospital of Traditional Chinese Medicine, 274 Zhijiang Middle Road, 200071 Shanghai, China; 3 School of Acupuncture-Moxibustion and Tuina, Shanghai University of Traditional Chinese Medicine, 1200 Cailun Road, 201203 Shanghai, China; 4 Shanghai Research Center of Acupuncture & Meridians, 274 Zhijiang Middle Road, 201203 Shanghai, China

**Keywords:** Diabetic peripheral neuropathy, Traditional Chinese medicine, Laser moxibustion, Nerve conduction velocity

## Abstract

**Background:**

Diabetic peripheral neuropathy (DPN) is the most common chronic neurological complication. It is the main cause of disability in diabetes mellitus (DM) patients and seriously affects the quality of life of patients. Pharmacological treatments always associate with limited efficacy and adverse effects. Moxibustion has been recommended to treat DPN as an adjuvant therapy to conventional medical treatment to accelerate alleviation of the symptoms of DPN. 10.6-μm laser moxibustion (LM), whose wavelength is close to the peak of infrared radiation spectrum of the traditional moxibustion as well as human acupoints, produces the thermal effect similar with moxibustion but with no smoke or smell. The purpose of this sham controlled clinical trial is to determine the effect and safety of 10.6-μm LM as adjuvant therapy in patients with DPN.

**Methods:**

This is a protocol for a randomized, double-blind, sham-controlled trial. One hundred fourteen patients meeting the inclusion and exclusion criteria will be recruited and randomly assigned to the LM group or the sham LM group with a 1:1 allocation ratio. Patients in both groups will receive a basic integrated treatment of Chinese and Western medicine and a total of 12 sessions of true or sham LM treatments over 4 weeks with 3 sessions a week. The primary outcome is nerve conduction velocity (NCV), and the secondary outcomes include Michigan Neuropathy Screening Instrument (MNSI) scores, Diabetes-Specific Quality of Life (DSQL) scores, blood rheology parameters, and assessments of safety and blinding. Outcome measures will be collected at baseline, 2 weeks after treatment, the end of LM treatments (4 weeks), and 4, 8 weeks after the end of LM treatment (8, 12weeks).

**Discussion:**

This study will be conducted to compare the efficacy of LM versus sham LM combined with medical treatment. 10.6-μm LM may alleviate symptoms, improve quality of life, and reduce the dosage of drugs as well as avoid causing serious side effects.

**Trial registration:**

Chinese Clinical Trial Registry ChiCTR2000029329. Registered on 25 January 2020.

## Background

Diabetes mellitus (DM) is a chronic systemic metabolic disease caused by a variety of reasons. According to an estimate by the International Diabetes Federation (IDF) in 2015, there were 415 million adults worldwide suffering from DM, about 8.8% of the adult population, and the figure was expected to exceed 600 million by 2040 [1]. In addition to the harm of diabetes itself, the chronic complications caused by diabetes are also the primary reasons affecting patients’ life quality and life expectancy. Diabetic peripheral neuropathy (DPN) is the most common chronic neurological complication of DM; the prevalence of DPN among DM patients is about 30~50% [2–5]. DPN develops as a consequence of long-standing hyperglycemia [6]. The mechanisms of DPN mainly involves activation of polyol pathway, advanced glycation end products (AGEs), dyslipidemia, oxidative stress, and lack of neurotrophic factors [7–9]. The main clinical manifestations of DPN are numbness or pain in both extremities, as well as paresthesia such as formication or burning sensation. DPN plays a key role in the development of diabetic foot complications [6]. About 15–20% of diabetic foot ulcers require amputation [10, 11]. More than 60% of the patients may suffer from sleep disorders caused by diabetic peripheral neuropathy pain (DPNP), which even may lead to anxiety, depression, and other mental disorders [12]. In addition, DPN is associated with skeletal muscle deficits such as neurogenic muscle atrophy and myasthenia [13]. DPN is the main cause of disability in DM patients and seriously affect the quality of life of patients. Additionally, DPN is associated with a huge human and economic burden on both the patients and the health care system [14, 15]. In addition to drugs that control glucose (such as insulin), α-lipoic acid, mecobalamin, nimodipine, angiotensin-converting enzyme (ACE) inhibitors, epalrestat, and some medicine for pain management such as anti-convulsants, and tricyclic antidepressant drugs are commonly used to treat DPN [5]. But theses medicines always associate with limited efficacy and adverse effects as well as higher costs [16–19].

Non-pharmacological treatments have also been proposed to treat DPN combined with pharmacological treatments to accelerate regeneration of the injured nerve. Moxibustion, a traditional Chinese medicine method with moxa burning over the acupuncture points, has been recommended to treat DPN [20]. Some clinical trials showed that moxibustion had positive effects on nerve conduction velocity (NCV) and clinic symptoms caused by DPN [21–23]. However, the burning moxa produces an annoying smoke and smell, which is irritating and might be harmful to both the patients and practitioners and might limit the use of moxibustion in the clinic [24, 25]. Like moxibustion, low level laser therapy (LLLT) is one of the common non-pharmacological treatments for DPN [26]. LLLT may produce biological stimulation to the nervous system [27]. CO_2_ laser is a far-infrared laser with the wavelength of 10.6 μm, which is close to the peak of infrared radiation spectrum of the traditional moxibustion and human acupoints [28, 29]. In addition, CO_2_ laser can be absorbed within 0.2 mm of the epidermis and produce a fast, marked, and lasting thermal effect on the skin surface like traditional moxibustion [30, 31]. We have developed a laser moxibustion (LM) device of 10.6 μm wavelength, which has the thermal nature of moxibustion without smoke and smell. Our previous studies showed that 10.6-μm LM may be effective in alleviating the symptoms of knee OA [32–34]. We also found that the 10.6-μm LM and the traditional moxibustion may result in similar effects in treating knee OA [35]. Besides, animal experimental results showed 10.6-μm LM can alleviate the neuropathic pain induced by oxaliplatin and increase the blood perfusion of the skin. However, there is no high-quality clinical research to evaluate the effects and safety of 10.6-μm LM in treating DPN.

The purpose of this placebo controlled clinical trial is to determine the effect of 10.6-μm LM on motor and sensory nerve conduction velocity (NCV), symptoms (clinical scores) and safety in patients with DPN.

## Methods/design

### Trail design

This is a randomized, double-blind, placebo-controlled trial to compare the effectiveness and safety of 10.6-μm LM with sham laser control in treating DPN. This trial will be conducted in the endocrine department of Shanghai Municipal Hospital of Traditional Chinese Medicine.

This trial has been approved by the Institutional Review Board (IRB) of Shanghai Municipal Hospital of Traditional Chinese Medicine (Approved No.: 2019SHL-KY-37). Trial registration: Chinese Clinical Trial Registry, ChiCTR2000029329. Registered 25 January 2019—retrospectively registered, http://www.chictr.org.cn/edit.aspx?pid=48490&htm=4

**Fig. 1:**
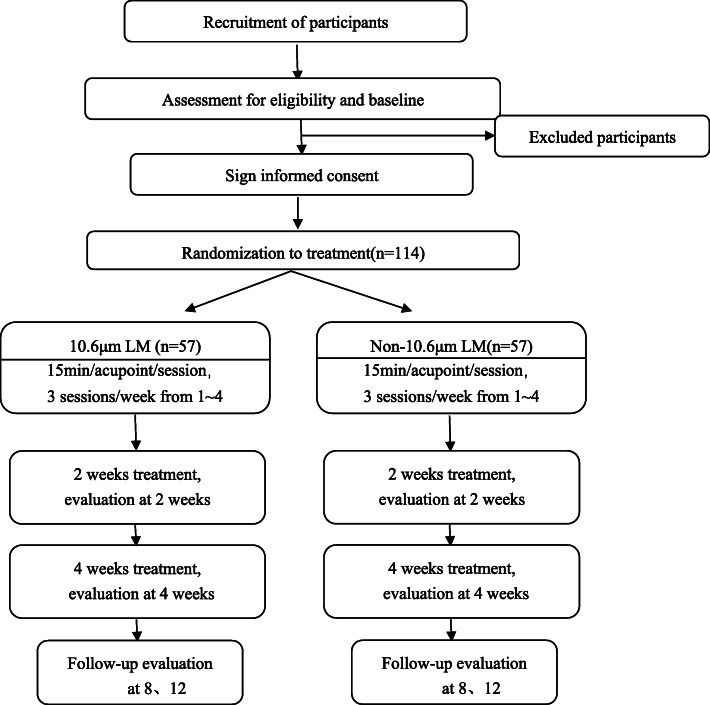
Study flow chart

Eligible patients will be randomly assigned into 2 groups (the LM group or the sham LM group) with a 1:1 allocation ratio. The patients in both groups will receive a total of 12 sessions of LM treatments over 4 weeks with 3 sessions a week, and the treatment will be given once every other day and lasts 15 min for each acupoint for each session. The outcomes will be measured at baseline, midterm (2 weeks after treatment), the end of LM treatment (4 weeks after treatment), and follow-up (4 、8 weeks after the end of LM treatment) (Fig. 1).

### Sample size

According to the studies [36, 37], we assume that the peroneal motor nerve conduction (MNCV) velocity in the LM group increases by 38% after treatment and that in the sham LM group by 16% after treatment. Fifty-one patients per group will be required with an alpha of 0.05 and a power of 0.8. Considering possible drop-out (i.e., 10% drop-out) during the trial, we will recruit 57 patients for each group.

### Study population

#### Inclusion criteria

1. Diagnosed with DPN [38] in both lower extremities
2. Aged 18–75 years old, no limit on gender
3. Delayed nerve conduction velocity in lower extremities
4. Stable blood glucose levels (Stable levels of HbA1c during the last 6 months)
5. Understanding and signing the informed consent

#### Exclusion criteria

1. Neuropathy or chronic pain caused by conditions other than diabetes
2. DPN most prevalent in the upper limbs
3. Presence of serious medical conditions including kidney diseases, heart diseases, pulmonary diseases, liver diseases or contagious diseases, or malignant tumors and serious psychopathy
4. Previous history of knee/hip replacement surgery or bone fracture of the lower extremities during the last 3 months and other conditions that would confound assessment of neuropathy
5. Ulceration and other diseases at the lower extremities
6. Acupuncture or moxibustion treatment received in the previous 3 months
7. LLLT received in the previous 3 months
8. Using other external therapy simultaneously
9. Opiate, analgesic, illicit drug, or alcohol abuse
10. Unwillingness to be randomly assigned into either a treatment or a placebo group
11. Unable to fill measurement questionnaires
12. Recruited in other clinical trial simultaneously
13. Pregnant or breast-feeding women

#### Withdrawal criteria

1. Serious adverse events during the treatment
2. Withdrawal demanded by the participant
3. Use the treatment method prohibited by the program or change the treatment method by the participant

#### Principles of management of withdrawal

1. Record the last treatment time and complete the evaluation items that can be completed
2. Intention analysis was performed on all cases after the end of the trial

### Randomization and blinding

The researcher will screen the patients strictly according to the inclusion and exclusion criteria. Eligible patients will be randomized in a ratio of 1: 1 into two groups: LM group or the sham LM group. The random assignment sequence will be generated by the researcher A who does not participate in other processes of the trial using SPSS software.

LM devices (either active or sham devices) will use different numbers as codes. The random assignment sequence will be placed in a serially numbered, opaque, sealed envelope with a copy paper inside by researcher A. Researcher B who will enroll participants will open the envelope in sequence after writing the basic information of the patient on the envelope surface before opening the envelope and informs the device operators of the device code assigned to the patient. Active or sham devices will be operated by trained operators separately.

The sham LM device is identical with the LM device in appearance, and operational procedure. The patients will be informed as follows in consent before randomization: “You will receive either 10.6 μm LM or non-10.6 μm LM treatment. You might feel any sensations (e.g., heat, cold, no feeling, or else) from this device. However, neither of the two methods can guarantee the curative effect…I have been told that I may be assigned randomly in 10.6 μm LM group or non-10.6 μm LM group [39].” Besides, the two groups of patients will be treated in separate rooms or treatment time to avoid communication among patients. Therefore, patients, researcher screening patients, device operators, outcome assessors, data entry researchers, and statistical personnel will be blinded to the treatment allocation.

### Consent

Before randomization, the patients will be informed the whole procedure of the trail and possible benefits or side effects by the researcher B. And then, written consent will be obtained from each participant.

### Intervention

#### Laser moxibustion device and sham device

**Fig. 2:**
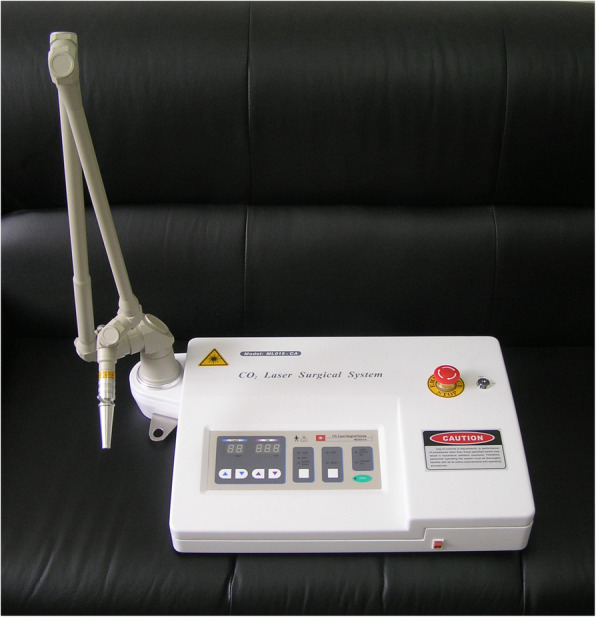
The LM devices (SX10-C1)

The LM devices (SX10-C1) were manufactured by Shanghai Wonderful Opto-Electrics Tech. Co., Ltd. (Shanghai, China) and licensed by Shanghai Municipal Food and Drug Administration, China (20162210783) (Fig. 2). The wavelength of the infrared laser is 10.6 μm; the output power is set between 140 and 160 mW. The sham LM device is identical with the LM device in appearance, weight, sound, and operational procedure but without laser output. However, in both active and sham devices, a red light-emitting diode with an output of 3 mW is used as visible indicator light on the skin to confirm accuracy of irradiation on the targeting acupoint.

#### Treatment protocol

Patients in both groups will receive LM or sham LM therapy and a basic concurrent therapy.

##### Laser moxibustion therapy

The patients will be told to lie down on their backs with the subknee area exposed entirely. The irradiation tips of the two LM devices will be aimed to the surface of the acupuncture points. The acupuncture points that are irradiated are the bilateral ST36 (*Zú Sān Lǐ*), GB34 (*Yáng Líng Quán*), SP6 (*Sān Yīn Jiāo*), and KI3 (*Tài Xī*) at the lower limbs. The location of all acupoints refers to the textbook *Acupuncture and Moxibustion* [40] (Table 1). The distance between the tips and the skin is about 2 cm, and the light spot is 2 cm in diameter on the skin. Each acupuncture point will receive 15 min irradiation of the LM device or sham LM device for one treatment. Energy density ranges from 40.13 to 45.86 J/cm^2^ for one session of treatments. The patients in both groups will receive a total of 12 sessions of treatments over 4 weeks with 3 sessions a week, and the treatment will be given once every other day. If the participants cannot participate a treatment session on schedule, he/she will be required to make it up within that week. The sham device is operated in the same way but there is no laser output.

**Table 1:** Acupuncture point selection

point	location
ST36(*Zú Sān Lǐ*)	On the anterior aspect of the leg, on the line connecting ST35 (*dú bí*) with ST41 (*jiĕ xī*), 3 cun inferior to ST35
GB34 (*Yáng Líng Quán*)	On the fibular aspect of the leg in the depression anterior and distal to the head of the fibula
SP6 (*Sān Yīn Jiāo*)	On the tibial aspect of the leg, posterior to the medial border of the tibia, 3 cun superior to the prominence of the medial malleolus
KI3 (*Tài Xī*)	On the posteromedial aspect of the ankle, in the depression between the prominence of the medial malleolus and the calcaneal tendon

##### Concurrent therapy

Patients in both groups will receive basic treatment such as blood sugar control according to patients’ condition. For the treatment of DPN, detailed recommended treatment plan is listed in Table 2. Any changes in the use of medications (including date of administration, types, and dosage) will be recorded in detail.

**Table 2:** Plan of the basic integrated treatment of Chinese and Western medicine

Medicine	Route of administration	Timing of administration
α-Lipoic acid 60 mg	Intravenous drip	Once a day from week 1 to week 2
Methycobal	Intravenous injection (1000 μg)	Once a day from week 1 to week 2
	Oral administration (500 μg)	Three times a day from week 3 to week 4
Epalrestat 50 mg	Oral administration	Three times a day from week 1 to week4

### Outcome measures

#### Primary outcome

##### Nerve conduction velocity (NCV)

NCV will be performed on both lower extremities at baseline and the week 4 (the end of treatment). Peroneal motor nerve conduction velocity (MNCV), sural sensory nerve conduction velocity (SNCV), and tibial motor nerve conduction velocity (MNCV) will be measured.

#### Secondary outcome

A. The most important secondary outcome is the Michigan Neuropathy Screening Instrument (MNSI) [41] scores at week 4 (the end of treatment). We will assess MNSI at baseline, week 2, week 8  and week 12. MNSI is comprised of the history questionnaire and the physical assessment. The history questionnaire is self-administered by the patients. Responses of “yes” to items 1–3, 5–6, 8–9, 11–12, and 14–15 are each counted as one point. A “no” response on items 7 and 13 counts as 1 point. Item 4 is a measure of impaired circulation, and item 10 is a measure of general asthenia and is not included in scoring [41]. The physical assessment comprises appearance of feet, ulceration, ankle reflexes, vibration perception at great toe, and monofilament and will be completed by a health professional.

B. Diabetes-Specific Quality of Life (DSQL) [42] will be used to assess health-related quality of life at baseline, week 4, week 8 and week 12. It mainly reflects the influence of diabetes and its treatment on the physiological, psychological, and social relations of patients. It has four dimensions: physiological function, psychological spirit, social relations, and the influence of treatment. DSQL has a total score of 120. The lower the score, the less affected by the disease and the better the quality of life.

C. Blood rheology parameters including whole blood low shear reduction viscosity, high shear reduction viscosity, plasma viscosity, and erythrocyte aggregation index will be measured at baseline and week 4.

D. Assessment of safety

We will collect any adverse event, whether related to treatment or not by the patients and practitioners at every session. During the 4-week follow-up period (from week 5 to week 12), we will telephone each patient every week to record any adverse event or side effect that occurs. Possible side effects of 10.6-μm laser moxibustion include redness and blisters. Serious adverse effects are reported to Medical Ethics Committee. The patients will answer questionnaire assessing the safety of the treatment at the end of LM treatment (week 4): “What do you think of the safety of laser therapy: safe, relatively safe, unsafe, and very unsafe” [39].

E. Blinding assessment

The patients in both groups will be asked to guess their group assignment allocation after the first treatment session and the end of treatment as following: “which group do you think you are in: A. 10.6-μm laser moxibustion; B. non-10.6-μm laser moxibustion; or C. not sure.” And the operators operating the true or sham LM devices will be also asked to answer the similar question.

J. Usage of medication

Medication usage log: The participants will be asked to record medications especially when changing the type or dosage compared with listed in Table 2.

The summarization of data collection plan is showed in Table 3.

**Table 3:** The summarization of data collection

	Baseline	Week 2	Week 4	Week 8 and Week 12(follow-up)
Demographic characteristics^a^	√	—	—	—
FPG,2hPBG, HbA1c	√	√	√	√
NCV	√	—	√	—
MNSI	√	√	√	√
DSQL	√	—	√	√
Blood rheology parameters	√	—	√	—
Assessment of safety	—	√	√	√
Blinding assessment	—	—	√	—
Intake of medicine	√	√	√	√

^a^ Demographic characteristics include age, sex, course of DM, course of DPN, BMI, and level of education

### Data management

The data will be double entered by two researchers who do not participate in other processes respectively.

We plan to hold mid-term report meetings twice a year to monitor patient safety and treatment efficacy data while the trial is ongoing.

### Statistical analysis

The statistical analysis will be performed using SPSS version 21.0 software. All continuous data that follow the normal distribution will be expressed as mean ± standard deviation (); data that do not conform to the normal distribution will be expressed as median (Q1, Q3). And categorical data will be expressed in terms of the number of cases. If the general numerical data follow normal distribution, then the independent sample *t* test will be used to analyze the differences between the two groups at different points in time. If the values do not follow normal distribution, then the Wilcoxon Mann-Whiney *U* rank sum test will be used. Chi-square test and Mann-Whitney *U* test will be used to analyze the categorical data. Differences are considered statistically significant at a *P* value less than 0.05.

## Discussion

DM is another major killer of human health in addition to malignant tumors and cardiovascular diseases. DPN, a common complication of DM, is the main cause of disability in DM patients and seriously affect the quality of life of patients. Drugs for improving microcirculation, correcting metabolic disorders and nourishing the nerves as well as for pain management are used to treat DPN. Because of their limited effectiveness on symptomatic relief and undesirable side effects, to find a convenient and effective therapy which can not only improve the symptom of DPN but also reduce the dosage of drugs and avoid the serious side effects is necessary. The 10.6-μm LM used in our research produces a thermal effect similar to that of traditional moxibustion but without smoke and smell. Besides, compared with the traditional moxibustion, the 10.6-μm LM is much easier and safer to operate and control with adjustable parameters. We can know the quantitative data of the therapy dosage that we can’t get if we use the traditional moxibustion therapy.

This is a double-blind, sham-controlled trial. The sham LM device is identical with the LM device in appearance, and operational procedure. To achieve successful masking, the two groups of patients are treated in separate rooms or treatment time, and the active or sham devices are operated by different trained operators. The participants and the devices operators are not informed explicitly that either true or sham laser treatment will be given. They are only informed that either 10.6-μm laser moxibustion or non-10.6-μm laser moxibustion will be given. The study design is a rigorous double-blind RCT, the arrangement of the trial process and the characteristics of the true and sham laser device make the blind method more reasonable.

The selected acupoints, ST36 (Zú Sān Lǐ), GB34 (Yáng Líng Quán), SP6 (Sān Yīn Jiāo), and KI3 (Tài Xī), are commonly used points for DPN [43]. These acupoints are located on the lower limbs and can help to replenish qi and the blood, warm the meridians, and improve blood circulation and disperse stasis through moxibustion.

In our study, we will use the nerve conduction study (NCS) as the primary outcome. NCS is also one of the objective measures in the diagnosis of DPN with high sensitivity and accuracy [44]. Besides, we will use the symptoms and examination outcome measurement (MNSI) to evaluate the severity of the symptom. The subjective outcome (DSQL) will be used to assess the influence of the treatment on patients’ quality of life. The blood rheology parameters will be used to assess the effect of 10.6-μm LM on improving blood rheology and decreasing blood viscosity in DPN patients.

There are two limitations of our study. Firstly, we focus more on the clinical effect of LLLT on DPN, not much attention has been paid to the possible mechanism of LLLT on DPN. Secondly, due to the limitations of the device, one treatment will last for 30 min, but each acupoint will be irradiated for 15 min.

A study showed that the increasing of NCV in the mecobalamin combined moxibustion group was higher than that in the mecobalamin alone group at the same endpoints [36]. Thus, we anticipate the 10.6-μm LM which shared similar infrared effect with the traditional moxibustion may result in similar curative effect and reduce the dosage of drugs as well as avoiding the serious side effects caused by western medicine. We expect this double-blind, sham-controlled trial will provide rigorous evidence for the effect and safety of 10.6-μm LM as adjuvant therapy in treating DPN.

## Trial status

The protocol version number is V1.0. Recruitment is expected to begin in May 2020, and the last patient is expected to be included in the study in May 2022.

## Data Availability

The results of this RCT trial will be published independently when this trail is completed. The full data set will be made available upon reasonable request and application for the data to be released should be made in contact to LL (study applicant).

## Abbreviations

DM: Diabetes mellitus
DPN: Diabetic peripheral neuropathy
DPNP: Diabetic peripheral neuropathy pain
LLLT: Low level laser therapy
LM: Laser moxibustion
NCS: Nerve conduction study
NCV: Nerve conduction velocity
MNCV: Motor nerve conduction velocity
SNCV: Sensory nerve conduction velocity
SNAP: Sensory nerve action potential
MNAP: Motor nerve action potential
MNSI: Michigan Neuropathy Screening Instrument
DSQL: Diabetes-Specific Quality of Life
RCT: Randomized controlled trial
FPG: Fasting plasma glucose
2hPBG: Two-hour plasma blood glucose
